# Study on Plastic Constitutive Relation and Ductile Fracture Criterion of AM60B Magnesium Alloy

**DOI:** 10.3390/ma17071684

**Published:** 2024-04-07

**Authors:** Qin Yang, Bin Jiang, Liang Gao, Yuyang Gao, Bin Liang, Sha Lan, Zeng Qin, Wenjun Zou, Fengying Yang, Fusheng Pan

**Affiliations:** 1 National Engineering Research Center for Magnesium Alloys, Chongqing University, No. 174, Shazheng Street, Shapingba District, Chongqing 400044, China; yangqin1@changan.com.cn (Q.Y.); gaoyuyang@cqu.edu.cn (Y.G.); lb20103312@163.com (B.L.); lansha1@changan.com.cn (S.L.); qinzeng00@163.com (Z.Q.); fspan@cqu.edu.cn (F.P.); 2 College of Materials Science and Engineering, Chongqing University, No. 174, Shazheng Street, Shapingba District, Chongqing 400044, China; 3 Changan Auto Global R&D Center, Changan Automobile Co., Ltd., No. 226 Liangjiang Road, Yuzui Town, Jiangbei District, Chongqing 401133, China; gaoliang@changan.com.cn (L.G.); changanzouwenjun@sina.com (W.Z.); angle1202yfy@163.com (F.Y.)

**Keywords:** AM60B, deformation behavior, stress states, JC (Johnson–Cook) fracture model, MMC (Modified Mohr–Coulomb Model) fracture model, DIEM (Damage Initiation and Evolution Model) fracture model

## Abstract

It is currently a challenge to accurately predict the deformation and fracture behavior of metal parts in automobile crashes. Many studies have shown that the deformation and fracture behavior of materials are significantly affected by the stress state during automobile crashes with complex stress state characteristics. In order to further promote the application of die-cast magnesium alloys in automobiles, it is particularly important to study the material deformation and fracture behavior of die-cast magnesium alloys. In this paper, the mechanical properties of the AM60B die-cast magnesium alloy sheet under four stress states (shear, tension, R10 notch tension, and cupping) were designed and tested. Based on the von Mises isotropic constitutive model and Swift weighted Hockett–Sherby hardening model, the plastic constitutive model of die-cast magnesium alloy was established. Based on the plastic model and the fracture model (JC, MMC, and DIEM) considering the influence of three stress states, the deformation and fracture behavior of the AM60B die-cast magnesium alloy front-end members in three-point bending were predicted by experiments and finite element simulation. The experimental results show that the deformation mode and loading–displacement curve trend of the AM60B die-cast magnesium alloy front members are the same, the crack initiation point and crack initiation time are the same, and the crack shape is similar. The results show that the complex stress state constitutive model parameters and the DIEM fracture model obtained in this paper can accurately predict the deformation and fracture failure behavior of the AM60B die-cast magnesium alloy sheet.

## 1. Introduction

Magnesium alloy is by far the lightest structural metal material, and due to its low density and high strength/weight ratio, it has wide application prospects in many fields, especially in the automotive and aircraft industries [1,2,3,4,5]. Due to its short processing cycle and assembly cost, cast magnesium alloys have significant economic advantages in the mass production of automotive parts [6,7,8,9,10,11]. The most widely used cast magnesium alloy in automobiles is die-cast magnesium alloy [12,13,14]. Currently, the large-scale application of die-cast magnesium alloy in automobiles mainly focuses on the steering wheel skeleton, car seat skeleton, and large display bracket of automobiles [15], which is not widely used. This is because magnesium alloys have extremely complex plastic deformation mechanisms and failure criteria, which have become some of the main obstacles to their wide application. The poor flexibility of magnesium alloy makes it easy to break in a collision, which becomes a major challenge for automobile safety.

Automobile collision is a dynamic process [16]. The nonlinear large deformation finite element simulation can help the designer accurately predict the deformation and fracture of magnesium alloy structure in the automobile design stage, so as to reduce the cost. Therefore, it is important to study the dynamic mechanical behavior of magnesium alloy materials and the plasticity and fracture models of magnesium alloy materials. The results show that magnesium alloys have strain rate sensitivity under tensile conditions. The fracture mechanism of metal materials is often ductile fracture, and its fracture strain is related to the stress state.

The methods to establish the constitutive relationship of metal materials can be divided into three types: the macroscopic phenomenological method, basic micromechanics method, and macromicroscopic combination method. In engineering applications, the constitutive relationship of materials is mostly established by macroscopic phenomenology. At present, the constitutive relations of materials established by macroscopic phenomenological method have been studied extensively in the world. Macroscopic phenomenological models include the Rice and Tracy fracture model [17], Bai–Wierzbicki model [18], DF series fracture criterion [19], JC (Johnson–Cook) fracture model [20], MC (Mohr–Coulomb fracture) model [21], MMC (Modified Mohr–Coulomb Model) fracture model [22], and DIEM (Damage Initiation and Evolution Model) fracture model [23]. JC, MMC, and DIEM are the most commonly used models of metal materials used in automotive engineering.

When it comes to high-precision simulation of the fracture behavior of materials, different researchers often choose different fracture models. Chen et al. [24] studied the constitutive behavior of large grain cast AZ80 considering only three directions of compression tests. The modified Arrhenius relation is used to reflect the constitutive behavior before the peak strain of compression test, and the modified Johnson–Cook model is designed to show the stage after the peak strain of compression test. Mirza et al. [25] also only considered compression tests to established a modified Johnson–Cook constitutive equation to predict the flow stress of extruded Mg-10Gd-3Y-0.5Zr (GW103K) magnesium alloy, and the standard deviation between the predicted results and the experimental results was approximately 1.8%. Zhigang Li et al. [26], using uniaxial tension for testing at different strain rates, put forward a rate-related Johnson–Cook modified model (M-J-C) to predict the plasticity and fracture behavior of AZ31B magnesium alloy at different strain rates. Aarjoo Jaimin et al. [27] used the Johnson–Cook and Zerilli–Armstrong constitutive models to predict the flow stress of AZ31B alloy at temperatures from 200 °C to 350 °C and strain rates from 10^−1^/s to 10^−3^/s.

Xu qing Chang et al. [28] studied the constitutive model of compression deformation of AZ80 magnesium alloy under various loading directions and strain rates, in which the log-M linear model was more accurate than the modified Johnson–Cook model. Jia et al. [29] replaced stress triaxiality and the Rhodes parameters in the MMC model with a strain ratio under the assumption of plane stress, established an eMMC model based on strain ratio, simplified the identification process of model parameters, and finally accurately predicted the fracture curve of advanced high-strength steel TRIP780. Li et al. [30] successfully predicted the initial exhaustion of aluminum alloy 6061 in multi-point progressive forming by using the MMC model. Xiao et al. [31] considering the influence of strain rate and temperature on material deformation behavior, extended the Modified Mohr–Coulomb Model (MMC) and used it to better predict the damage failure of aluminum alloy AA2024-T351 target plate under the impact of the projectile body. Based on the MMC criterion, Ji et al. [32] considered the effects of strain rate effect and anisotropy effect on the fracture behavior of AA6061-T5 aluminum alloy. Then, the strain rate correlation function and HILL4 anisotropic yield criterion were used to improve the MMC criterion, which can predict the ductile fracture behavior of AA6061-T5 at different strain rates. Du et al. [33] considered the effects of the strain rate and temperature on the fracture behavior of aluminum alloy AA5383, and introduced the correlation function of strain rate and temperature to extend the MMC model. In addition, the MMC model has also been applied in the prediction of ductile fracture behavior of magnesium alloy materials [34]. Ma Hongyue et al. [35] concluded that the MMC fracture model has good general suitability in aluminum and magnesium alloys with different forming processes after reviewing the literature.

The DIEM failure model divides the fracture modes of materials into positive ductile fracture, shear fracture, and necking instability failure, and its damage initiation submodel is defined as the critical strain related to the stress state corresponding to different fracture modes. Chunhua Tian et al. [36] studied the damage initiation and growth mechanism of two DP800 steels by studying different prestrain samples. Wang Dong et al. [37,38] established the DIEM failure model and GISSMO failure model of thermoformed B1500HS and DP780 dual-phase steel, respectively, and found that the DIEM failure model could better predict the failure behavior of the corresponding materials. HooPutra et al. [39,40,41,42,43], respectively, established the DIEM failure model of EN AW-7108 T6 aluminum alloy and applied it to the thin-wall energy absorption tube structure. It was found that the finite element model with the failure model added could better predict the tearing behavior of the energy absorption tube during axial collapse. Based on DIEM failure model, the thin-walled pipe is optimized.

Therefore, for the above research, whether it is the JC model, MMC model, or DIEM model, the study of plastic constitutive only considers single tension, unidirectional compression, high-temperature tension, etc. However, there is almost no research on constitutive models under complex stress states. At the same time, only scholars have studied the JC model for the fracture model of magnesium alloys, while there is less research on the other two types of models. However, the plastic constitutive and fracture types of die-cast magnesium alloys have not been studied. The most suitable process for magnesium alloys used in automobiles is die-casting. In order to further promote the application of die-cast magnesium alloy in automobile, this paper takes the AM60B die-cast magnesium alloy sheet as the research object and establishes constitutive models under four different stress states to accurately predict its deformation behavior. At the same time, based on JC, MMC, and DIEM, three fracture models considering stress states, the simulation prediction and test comparison of fracture behavior under four stress states are performed, and it is found that DIEM fracture model can accurately predict the fracture failure situation under each stress state. Based on this, the three-point bending simulation and test of front-end components have a good agreement, which provides design basis and theoretical support for the practical application of die-casting AM60B magnesium alloy in automobiles.

## 2. Experimental Procedures

### 2.1. Basic Mechanical Properties Test

**Figure 1:**
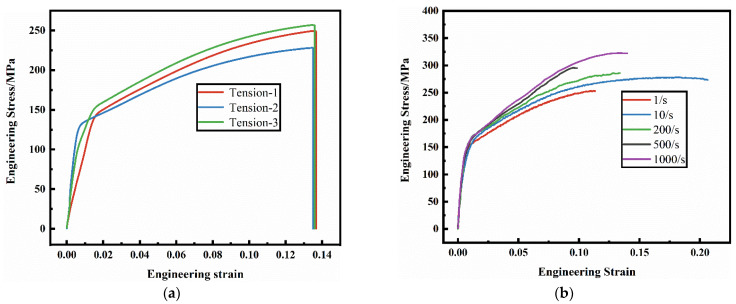
Engineering stress–engineering strain curve of the AM60B die-cast magnesium alloy sheet: (**a**) quasi-static tension; (**b**) dynamic tension.

The AM60B die-cast magnesium alloy sheet is used as a structural part in the automobile, facing the characteristics of the service environment from static to dynamic. In order to obtain the basic mechanical properties of the AM60B die-cast magnesium alloy, standard tensile specimen was taken on magnesium plate according to GB/T228 standard [44] and quasi-static uniaxial tensile test was performed. The results are shown in Figure 1a. It can be seen from the figure that the yield stress and ultimate tensile strength of the AM60B die-cast magnesium alloy are 143 MPa and 246 MPa, and there is no obvious local deformation after necking, and rapid fracture occurs. The mechanical properties of the AM60B die-cast magnesium alloy at strain rates of 1 s^−1^, 10 s^−1^, 200 s^−1^, 500 s^−1^, and 1000 s^−1^ were tested by high-speed tensile testing machine, and the results are shown in Figure 1b. It can be seen from the figure that the yield stress of the AM60B die-cast magnesium alloy increases with the increase of strain rate, indicating that its mechanical properties are sensitive to strain rate [45,46,47,48,49].

### 2.2. Ductile Fracture Test

It is pointed out that the plastic deformation behavior of metal materials is related to strain rate and stress state [50,51]. In the three-dimensional stress state, the stress state of the material is commonly characterized by stress triaxiality *η* and lode angle parameter *ξ* [52]. In the two-dimensional stress state such as plane stress, since the third principal stress is zero, the stress state of the material is characterized by only one parameter such as stress triaxiality *η* or lode angle parameter *ξ*. The stress triaxiality *η* and lode angle parameter *ξ* can be calculated by the following formula:

(1) $$\eta =\frac{\sigma_{m}}{\bar{\sigma }}=\frac{\frac{1}{3}(\sigma_{1}+\sigma_{2}+\sigma_{3})}{\bar{\sigma }}=\frac{\frac{1}{3}I_{1}}{\sqrt{3J_{2}}}$$

(2) $$\xi =\frac{27}{2}\frac{J_{3}}{{\bar{\sigma }}^{3}}=\frac{3\sqrt{3}}{2}\frac{J_{3}}{J_{2}{\;}^{3/2}}$$

where σ_m_ is the average stress. $\bar{\sigma }$ is the von Mises equivalent stress. σ_1_, σ_2_, and σ_3_ are the first, second, and third principal stress, respectively. I_1_ is the invariant of the first stress tensor. J_2_ and J_3_ are the invariant of the second and third deviatoric stress tensors, respectively.

**Figure 2:**
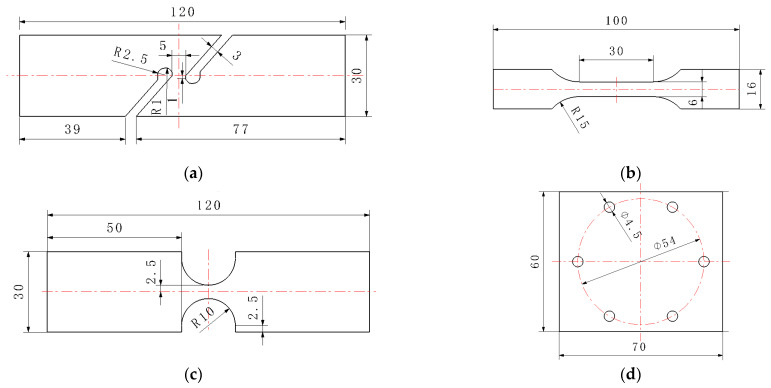
Sample size of the AM60B die-cast magnesium alloy sheet under different stress states (mm), (**a**) shear specimen; (**b**) unidirectional tensile specimen; (**c**) R10 notch tensile specimen; (**d**) cup specimen.

**Figure 3:**
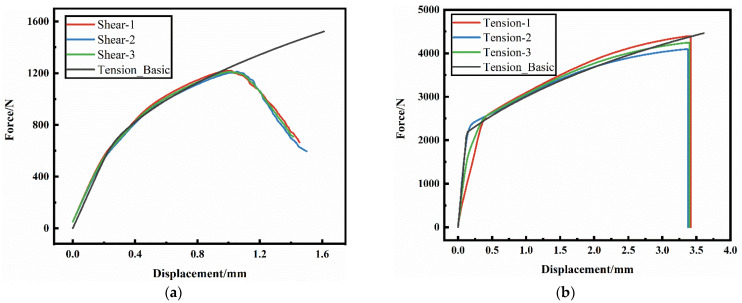

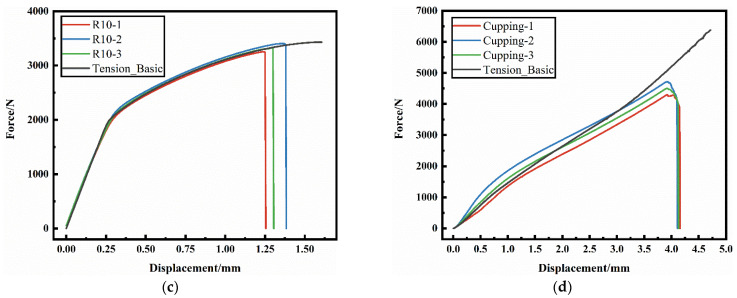
The loading–displacement curves of specimens under different stress states were tested: (**a**) shear test; (**b**) unidirectional tensile test; (**c**) R10 notch tensile test; (**d**) cupping test.

The AM60B die-cast magnesium alloy sheet, as a structural part in the automobile, also faces the service environment characteristics of complex stress states. In order to obtain the fracture properties of the AM60B die-cast magnesium alloy under different stress states, specimens under four stress states were designed for quasi-static test, namely the shear specimen, unidirectional tensile specimen, R10 notch tensile specimen, and cup specimen, as shown in Figure 2a–d. Shear, unidirectional tensile and R10 notch tensile tests were performed on the tensile testing machine. A DIC virtual extensometer with a length of 25 mm was used to test the loading–displacement curves of each test. The cupping test was performed by using the designed tool on the tensile testing machine. After the specimen is fixed by bolts, the semi-circular punch with a radius of 5 mm impacts the central position of the sample at a speed of 6 mm/min. When the affected area breaks, the test is stopped, and the contact force between the punch and the magnesium alloy sheet and the displacement curve of the punch are output. Each test was repeated for three times, and the loading–displacement curve obtained by the test is shown in Figure 3. In the shear test, obvious cracks began to occur at the moment of maximum force, which was the moment of crack initiation. It can be seen from the figure that under each stress state, the specimen breaks quickly after reaching the maximum force value, and there is no obvious local deformation phenomenon after all.

## 3. Results and Discussion

### 3.1. Plastic Constitutive Relation Study

In finite element simulation, constitutive model is commonly used to describe the elastic-plastic deformation behavior of sheet metal, which mainly includes yield criteria and hardening criteria. Yield criteria are used to describe when a material reaches the yield surface. Hardening criterion is used to describe the changes of yield loci during the deformation of a material [53]. It is pointed out that a magnesium alloy sheet is anisotropic, and its deformation behavior should be characterized by anisotropic constitutive model [54]. Considering engineering application scenarios, such as automobile magnesium alloy front end components, in vehicle collision simulation modeling, the material direction should be specified according to the direction of the raw materials and parts-forming process, and parts with different raw materials and different forming processes should be specified different material directions, which greatly reduces the modeling efficiency. Under the premise of both modeling efficiency and calculation accuracy, most of the isotropic constitutive models are used to simulate and analyze the deformation behavior of magnesium alloy parts in the automobile industry. Therefore, in this paper, the plastic constitutive relationship of the AM60B die-cast magnesium alloy was studied by using the isotropic constitutive model based on von Mises yield criterion [55].

#### 3.1.1. Study on Constitutive Relations under Uniaxial Stress

**Figure 4:**
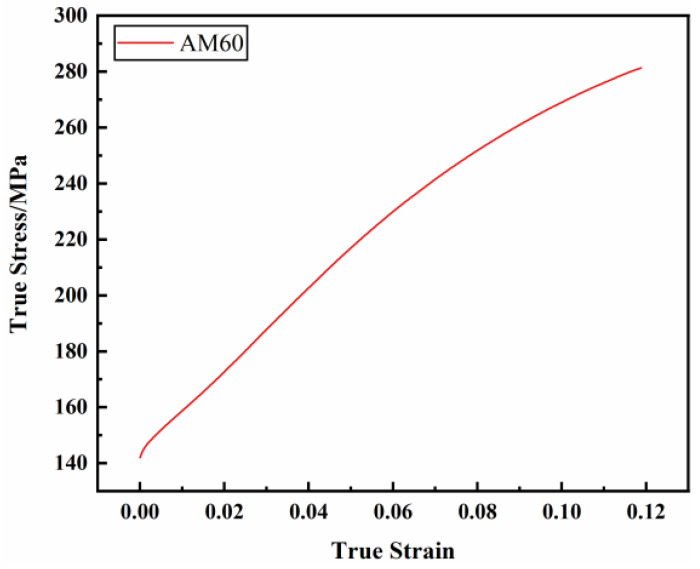
Quasi-static true stress–plastic strain curve of the AM60B die-cast magnesium alloy.

Under quasi-static conditions, the true stress–plastic strain curve of the AM60B die-cast magnesium alloy is shown in Figure 4. As can be seen from the figure, quasi-static unidirectional tensile test can only test the true stress of the AM60B die-cast magnesium alloy under 0–0.12 plastic strain. In order to predict the large deformation behavior of the AM60B die-cast magnesium alloy auto parts during service, the true stress–plastic strain curve should be extended by using a hardening model.

**Figure 5:**
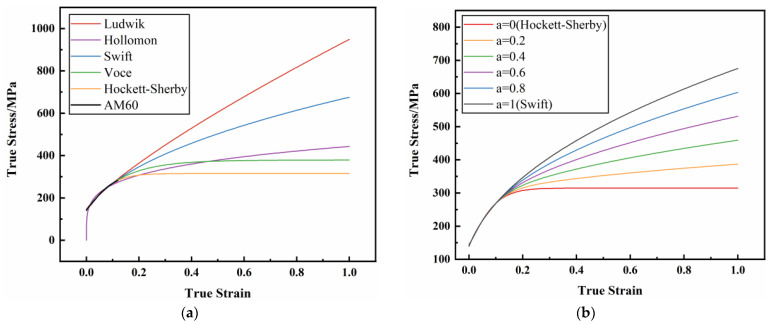
The true stress–plastic strain curve hardening model fits epitaxy: (**a**) different hardening models fit epitaxy; (**b**) fitting epitaxy with different weighting coefficients.

For metal materials, the commonly used hardening models include Ludwik, Hollmon, Swift, Voce, Hockett–Sherby, etc. [56,57,58,59,60], and the true stress–plastic strain curves fitted by different hardening models have great differences, as shown in Figure 5a. In order to increase the freedom of fitting the hardening model, the Swift weighted Hockett–Sherby hardening model method, as shown in Equation (3), was adopted in this paper to epitaxial the true stress–plastic strain curve. Different epitaxial true stress–plastic strain curves could be obtained by setting different weighting coefficient values, as shown in Figure 5b.

(3) $$\sigma_{T}=\alpha \cdot K\cdot {\left(\varepsilon_{0}+\varepsilon_{\mathrm{pl}}\right)}^{n}+(1-\alpha )\cdot (\sigma_{0}+A\cdot (1-e^{-B(\varepsilon_{\mathrm{pl}}{)}^{m}}))$$

where σ_T_ is the true stress. ε_pl_ is the plastic strain. α is the weighting coefficient, 0 ≤ α ≤ 1. And, K, ε_0_, n, σ_0_, A, B, and m are the unknown coefficients. Among them, parameter K is 666.769, ε_0_ is 0.02929, n is 0.444, σ_0_ is 146.361, A is 168.581, B is 23.815, and m is 1.263.

**Figure 6:**
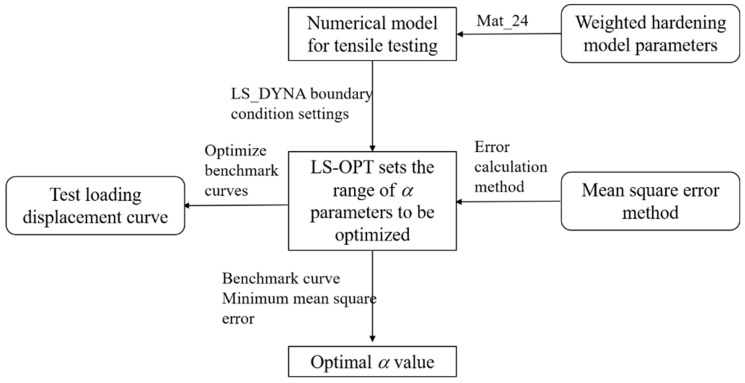
Parametric inverse process of weighted coefficient α under uniaxial stress state.

In order to determine the optimal weighting coefficient to accurately characterize the plastic deformation behavior of the AM60B die-cast magnesium alloy, based on the quasi-static unidirectional tensile test conditions and test results, the parameter inverse method optimized by experiment and simulation was adopted to optimize the weighting coefficient. The parameter inverse process is shown in Figure 6. Parameter inversion is performed in LS_OPT 7.0.0, and the optimal weighting coefficient is obtained by adjusting different weighting coefficients and comparing loading–displacement curves obtained by test and simulation calculation. Firstly, the true stress–plastic strain curve before the necking point is obtained based on the uniaxial tensile test of AM60 magnesium alloy. Assuming that the initial value of the weighting coefficient α is 0.5, the initial epitaxial true stress–plastic strain curve is obtained by using Equation (3) for fitting calculation. Secondly, according to the sample size and test conditions of the quasi-static uniaxial tensile test, the numerical model of the tensile test shell element is established in LS_PREPOST 4.8.0(the pre-processing software of LS_DYNA 11.2.2), in which the mesh type is TYPE16 and the mesh size of the parallel segment is 0.5 mm [61]. The dimensions and boundary conditions of the model are consistent with the experimental process, and the initial epitaxial true stress–plastic strain curve is added to the material constitutive model of LS_DYNA No. Mat_24. The established numerical model of tensile test is imported into LS_OPT software, and the weighting coefficient α is set from 0 to 1. The loading–displacement curves obtained by test and simulation are set as benchmarked curves, and the mean squared errors (MSE) method is used to calculate the coincidence degree of curves. Finally, LS_DYNA is selected as the solver in LS_OPT to optimize the calculation of the weighting coefficient α. The optimal weighting coefficient α is obtained when the mean square error value between loading–displacement curves is the lowest.

**Figure 7:**
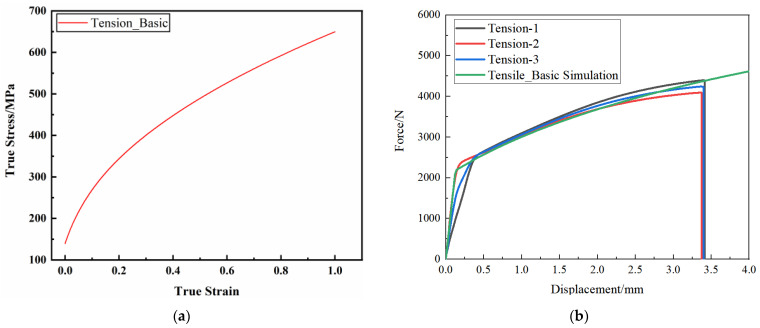
Inverse results of LS OPT parameters of uniaxial stress state constitutive model: (**a**) epitaxial true stress–plastic strain curve; (**b**) benchmarking results of loading–displacement curves.

The optimal weighting coefficient obtained by parameter inversion is 0.2, and the corresponding epitaxial true stress–plastic strain curve is shown in Figure 7a, which is the plastic constitutive model parameter of the AM60B die-cast magnesium alloy under uniaxial stress state. The loading–displacement curve calibration results are shown in Figure 7b. It can be seen from the figure that the curve trends of the test and simulation are the same, with a high degree of coincidence, and the mean square error between the curves is 2%.

**Figure 8:**
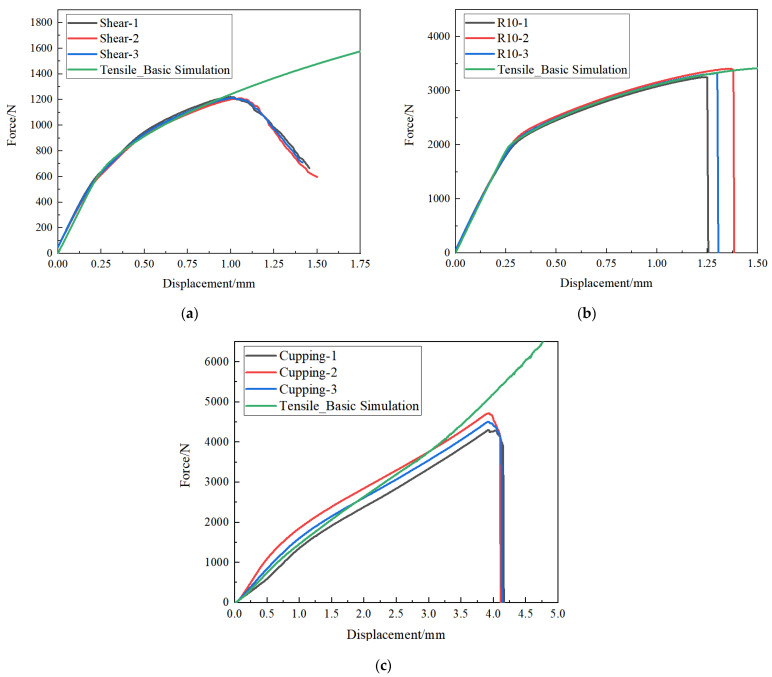
The benchmarking results of loading–displacement curves of tests under different stress states: (**a**) shear test; (**b**) R10 notch tensile test; (**c**) cupping test.

In order to verify the applicability of the parameters of the plastic constitutive model, it was used to predict the plastic deformation behavior of the AM60B die-cast magnesium alloy under three stress states: shear, R10 notch tensile, and cupping. Through calculation, the comparison of loading–displacement curves between test and simulation under various stress states is shown in Figure 8. The relative error between maximum forces and the maximum relative error under the same displacement is shown in Table 1, where the relative error between maximum forces is the error between the tested maximum force and the simulated maximum force before the test fracture. The maximum relative error of the force under the same displacement is the maximum error between the load forces under the same displacement.

It can be seen from Figure 8 and Table 1 that the epitaxial true stress–plastic strain curve optimized by unidirectional tensile test has low accuracy in predicting the deformation behavior of the AM60B die-cast magnesium alloy sheet under other stress states, especially in the cupping test, where the error is greater than 10%. The main reason for the analysis is that the influence of other stress states on the weighting coefficient is not considered when the one-way tensile test is only used to optimize the weighting coefficient, that is, the weighting coefficient is only the local optimal value rather than the global optimal value.

#### 3.1.2. Study of Constitutive Relations under Complex Stress States

**Figure 9:**
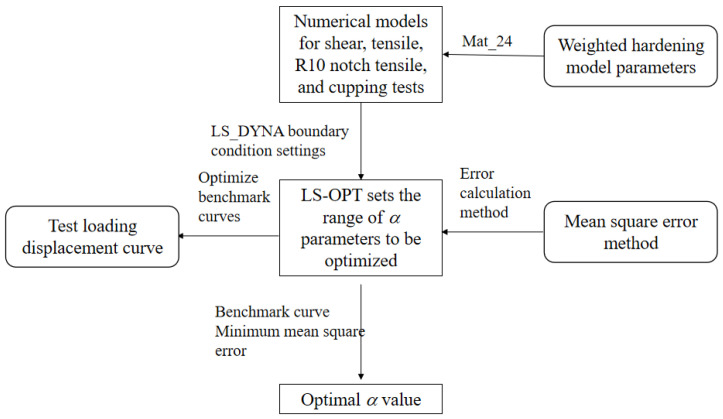
Inverse flow of parameters of weighted coefficient α under complex stress state.

In order to obtain the constitutive relationship of the AM60B die-cast magnesium alloy sheet under complex stress states, the weighted coefficient α was optimized by using shear, tensile, R10 notch tensile, and cup samples. The mean square error between the test and simulation loading–displacement curves under the four stress states was the lowest, and the globally optimal weighted coefficient value was output, as shown in Figure 9.

The optimization process of α under complex stress is similar to that of unidirectional tensile stress. The numerical models of shell elements under four stress states of shear, tensile, R10 notch tensile, and cup process should be established in LS_DYNA 11.2.2 software at the same time, in which the mesh type is TYPE16, and the mesh size in the main deformation area is 0.5 mm. The dimensions and boundary conditions of each model are consistent with the experimental process, and the material constitutive model Mat_24 of LS_DYNA is adopted.

**Figure 10:**
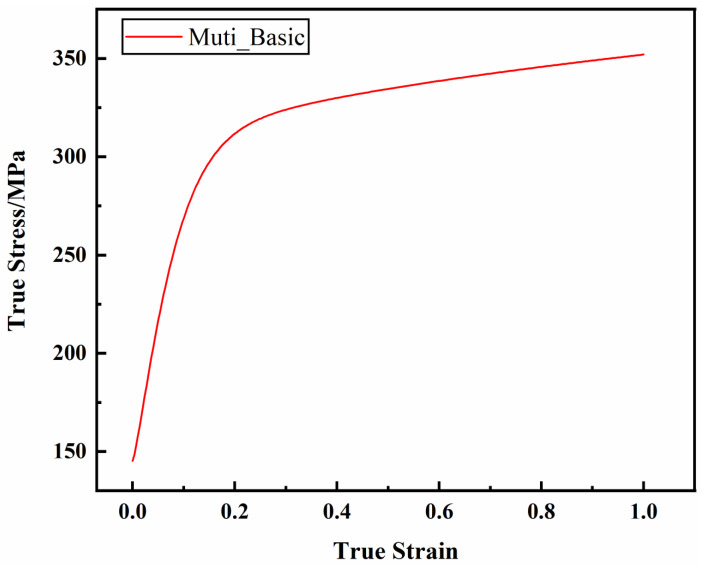
The weighted coefficient epitaxial the true stress–plastic strain curve.

**Figure 11:**
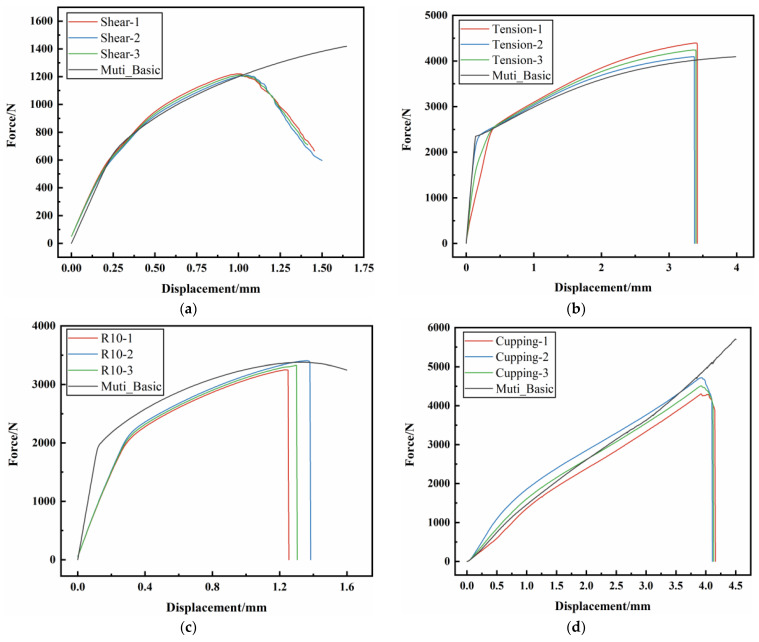
The benchmarking results of loading–displacement curves of each sample: (**a**) shear test; (**b**) unidirectional tensile test; (**c**) R10 notch tensile test; (**d**) cupping test.

The global optimal weighting coefficient α obtained by parameter inversion is 0.185, and the corresponding epitaxial true stress–plastic strain curve is shown in Figure 10, which is the plastic constitutive model parameter of the AM60B die-cast magnesium alloy under complex stress state. The comparison of loading–displacement curves between test and simulation under various stress states is shown in Figure 11, and the error between curves is shown in Table 2. As can be seen from the figure, the trend of loading–displacement curves of the test and simulation under each stress state is the same, with a high degree of agreement.

By comparison with Figure 8 and Figure 11, and Table 1 and Table 2, it can be seen that when the weighting coefficient α is globally optimized by using multiple stress state tests, the obtained epitaxial true stress–plastic strain curve has a higher overall accuracy in characterizing the deformation behavior of the AM60B die-cast magnesium alloy sheet under complex stress states. The error is increased by 3% in the tensile stress state, reduced by 3% in the shear stress state, and reduced by more than 5% in the R10 and cup stress states. Compared with the two methods of hardening curve optimization, the latter has higher comprehensive accuracy. It can be seen that the global optimization method for the weighting coefficient by using multiple stress state tests can increase the accuracy of the deformation behavior characterization of the material model for multiple stress states.

#### 3.1.3. Study on Dynamic Constitutive Relations

**Figure 12:**
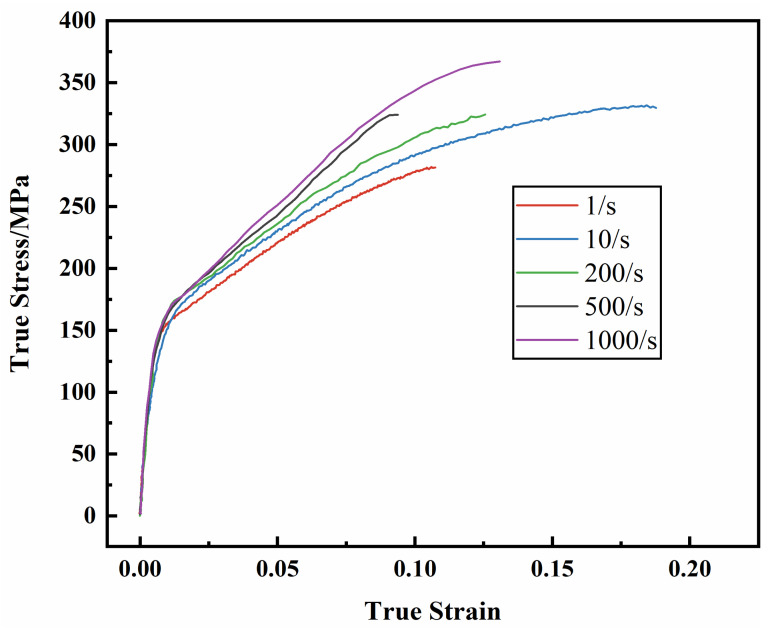
True stress–strain curves under different strain rates.

According to the high-speed tensile results (Figure 1), the tensile strength of AM60B magnesium alloy sheet increases with the increase of strain rate, indicating that strain rate has an effect on the deformation behavior of AM60B magnesium alloy sheet. The true stress strain at high strain rate is shown in Figure 12.

Strain rate strengthening effect refers to the phenomenon that deformation resistance of materials increases with the increase of strain rate in the process of plastic deformation, which belongs to the strengthening effect when materials undergo plastic deformation [62]. For most automotive metal sheets, there is strain rate strengthening effect. In the constitutive model, Johnson–Cook equation [63], modified Johnson–Cook equation [64] and Cowper–Symonds equation [65] are often used to consider the strain rate strengthening effect of sheet metal. In the Johnson–Cook equation, the equivalent stress is a function of plastic strain, strain rate and temperature. For automotive metal sheets, the temperature term in the Johnson–Cook equation is often not considered, that is, the Johnson–Cook equation is simplified to Equation (4), as follows:

(4) $$\bar{\sigma }=\left(A+B\varepsilon_{p}^{n}\right)\left[1+C\ln\left(\frac{\varepsilon_{p}}{\overset{\cdot }{\varepsilon_{p}^{0}}}\right)\right]$$

**Figure 13:**
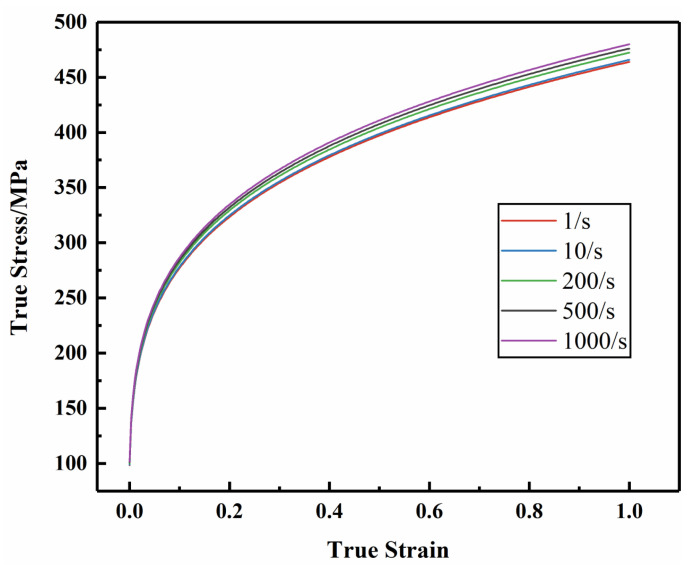
Dynamic constitutive relationship curve of the AM60B die-cast magnesium alloy sheet.

In the Johnson–Cook equation, parameters A, B, C, and n can be obtained by fitting the true stress–true strain curves and yield strength values of materials at different strain rates. The revised Johnson–Cook equation is obtained by modifying the Johnson–Cook equation, as shown in Equation (5), as follow:

(5) $$\bar{\sigma }=B\varepsilon_{p}^{n}\left[1+C\varepsilon_{p}^{n^{\prime }}\ln\left(\frac{\overset{\cdot }{\varepsilon_{p}}}{\overset{\cdot }{\varepsilon_{p}^{0}}}\right)\right]$$

where, B, C, and n are the parameters to be determined. In the Cowper–Symonds equation, the yield strength of materials under different strain rates can be solved by quasi-static yield strength, as shown in Equation (6):

(6) $$\bar{\sigma }=\left[{\left(1+\frac{\overset{\cdot }{\varepsilon_{p}}}{C}\right)}^{\frac{1}{p}}\right]\sigma_{0}$$

where, $\sigma_{0}$ is the yield stress under quasi-static conditions, and C and P are the parameters to be determined. When the strain rate effect is considered in the Cowper–Symonds equation, the values of the C and P parameters can be obtained by fitting the yield stress value of the material under different strain rates. The fitting degrees of the three strain rate equations at each strain rate are shown in Table 3. The curve obtained by fitting Cowper–Symonds equation with the highest fitting degree is selected as the dynamic constitutive relation curve of the AM60B die-cast magnesium alloy sheet, as shown in Figure 13.

### 3.2. Study on Fracture Criterion under Complex Stress State

The fracture behavior of metal is related to the stress state, and the stress state of metal plates is commonly characterized by the stress triaxial degree. In shear, tensile, R10 notch tensile, and cupping tests, the stress state of each specimen cannot be directly measured by the test. In addition, when DIC is used to track and test the strain information, the strain information obtained by direct test is full strain, and it is difficult to accurately measure the critical fracture equivalent plastic strain.

In order to obtain the stress triaxiality and critical fracture equivalent plastic strain of each sample, based on the size of each sample and the test process, the complex stress state constitutive model parameters were used to simulate and reproduce each test process. The element information of the main deformation region of each specimen in the simulation was extracted, the maximum equivalent plastic strain corresponding to the fracture time was taken as the critical fracture equivalent plastic strain of each specimen, and the stress state of each specimen was characterized by the mean value of the stress triaxial degree of the corresponding element of the critical fracture equivalent plastic strain, as shown in Table 4. It can be seen from the table that the critical fracture equivalent plastic strain values corresponding to the stress states of each specimen are different, indicating that the stress states have a great influence on the fracture behavior of the AM60B die-cast magnesium alloy.

**Figure 14:**
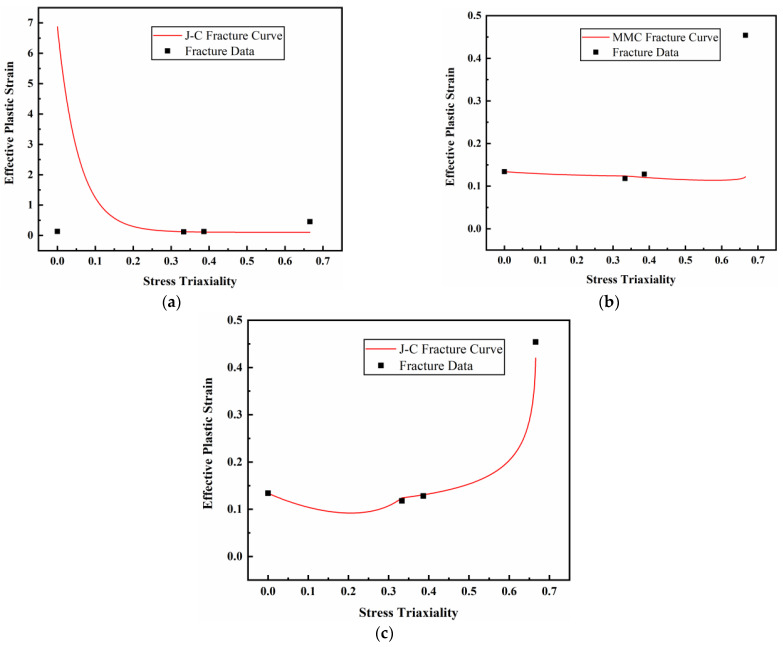
Each fracture model fits the curve: (**a**) JC fracture model curve; (**b**) MMC fracture model curve; (**c**) DIEM fracture model curve.

Based on the data in Table 4, the JC, MMC, and DIEM fracture models were used for fitting, as shown in Equations (7)–(9), and the fitting parameters and fracture curves of each model were obtained, as shown in Table 5 and Figure 14, respectively. According to the fitting results, the DIEM has the best fitting effect and the highest fitting correlation coefficient, which is 0.9999.

The JC fracture model is based on the theory of hole growth, which considers the effects of stress triaxial degree, strain rate, and temperature on the fracture properties of materials. Under normal temperature and quasi-static conditions, the fracture model is shown in Equation (7), as follows:

(7) $$\varepsilon_{f}\left(\eta ,\overset{\cdot }{\varepsilon },T\right)=\left[D_{1}+D_{2}e^{\left(D_{3\eta }\right)}\right]\left[1+D_{4}\ln(\frac{\overset{\cdot }{\varepsilon_{p}}}{\overset{\cdot }{\varepsilon_{p}^{0}}})\right]\left[1+D_{5}(\frac{T-T_{r}}{T_{m}-T_{r}})\right]$$

where, D_1_, D_2_, and D_3_ are the material parameters and are the critical fracture equivalent plastic strains of the material under different stress states. The MMC fracture model takes into account the influence of stress triaxiality and lode angle parameters, as shown in Equation (8):

(8) $$\varepsilon_{f}(\eta ,\xi )={\left\{\frac{K}{C}\left[C_{\theta }^{s}+\frac{\sqrt{3}}{2-\sqrt{3}}(1-C_{\theta }^{s})(\sec(\frac{\pi \bar{\theta }}{6})-1\right]\left[\sqrt{\frac{1+f^{2}}{3}}\cos(\frac{\pi \bar{\theta }}{6})+f(\eta +\frac{1}{3}\sin(\frac{\pi \bar{\theta }}{6}))\right]\right\}}^{-\frac{1}{n}}$$

where, $\varepsilon_{f}(\eta ,\xi )$ is the critical fracture equivalent plastic strain of the material under different stress states, and K, C, f, and n are the material parameters. The DIEM fracture model considers the influence of stress state on the fracture properties of materials, as shown in Equation (9):

(9) $$\varepsilon_{f}(\eta ,\tau_{\max})=e_{0}\cdot e^{\left(-f\theta \right)}+e_{1}\cdot e^{\left(f\theta \right)}\theta =\frac{1-k\eta }{\omega }\omega =\frac{\tau_{\max}}{\bar{\sigma }}$$

where, k, f, $e_{0}$, and $e_{1}$ are the material parameters, $\tau_{\max}$ are the maximum shear stress, $\bar{\sigma }$ are the equivalent stress, and $\varepsilon_{f}(\eta ,\tau_{\max})$ are the critical fracture equivalent plastic strain of the material under different stress states.

**Figure 15:**
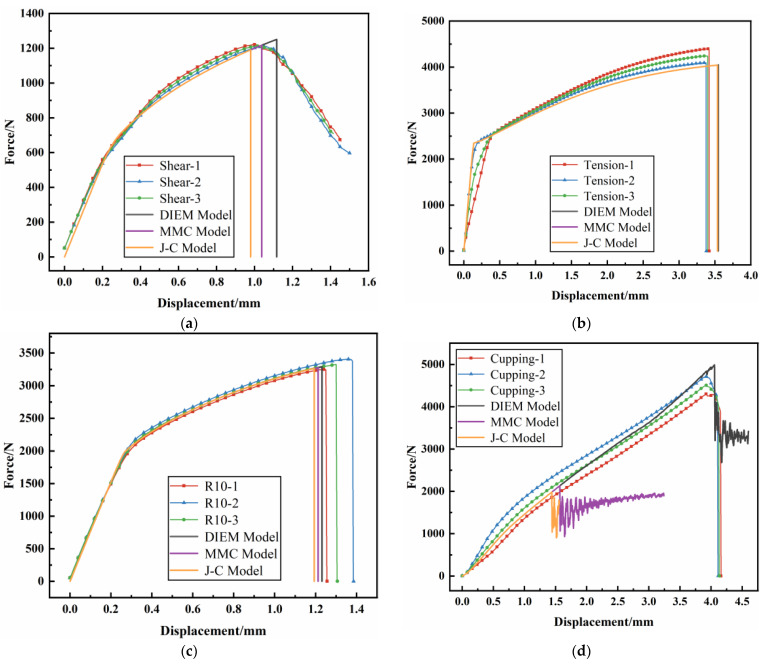
Three kinds of fracture predict the loading–displacement curves of specimens under different stress states: (**a**) shear test; (**b**) unidirectional tensile test; (**c**) R10 tensile test; (**d**) cupping test.

In order to verify the prediction accuracy of fracture model parameters on the fracture behavior of the AM60B die-cast magnesium alloy, simulation models of stress state tests were established. The JC, MMC, and DIEM fracture models were, respectively, used to predict the fracture behavior of the AM60B die-cast magnesium alloy, and the comparison results were shown in Figure 15 and Table 6. It can be seen that different fracture models have different accuracy in predicting the fracture time of each stress state. Under shear stress, the fracture time errors of the JC, MMC, and DIEM fracture models are 9.28%, 3.86%, and 3.39%, respectively. Under tensile stress, the fracture time errors of the JC, MMC, and DIEM fracture models are 4.05%, 4.46%, and 4.27%, respectively. Under R10 notch tensile stress, the fracture time errors of the JC, MMC, and DIEM fracture models are 28.11%, 26.31%, and 25.18%, respectively. Under cupping stress, the fracture time errors of the JC, MMC, and DIEM fracture models are 63.84%, 60.30%, and 2.40%, respectively. Under the stress state, the comprehensive error of fracture time predicted by DIEM fracture model is the smallest, which is 4.15%, indicating that the DIEM fracture model has a high accuracy in predicting the fracture behavior of the AM60B die-cast magnesium alloy.

### 3.3. Application of Constitutive Model and Fracture Model

In order to further verify the accuracy and applicability of the complex stress state constitutive model and fracture model parameters obtained by the research, a quasi-static three-point bending numerical model was established in LS_DYNA to simulate and predict the deformation and fracture behavior of the AM60B die-cast magnesium alloy under three-point bending conditions.

**Figure 16:**
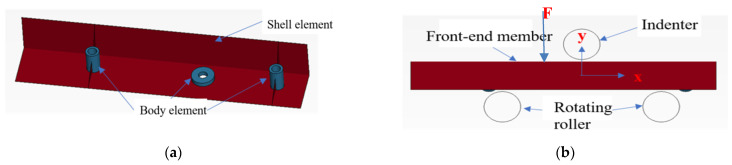
The three-point static simulation model of front-end component is established: (**a**) front-end component model; (**b**) three-point static simulation model.

The established three-point bending numerical model of the AM60B die-cast magnesium alloy front-end member is shown in Figure 16. The dimension information of the front-end member is obtained according to the actual structure measurement, which consists of the plate area, two high cylinders and one low cylinder structure. The plate (red area in the figure) adopts a shell element with a size of 2 mm, and the cylinder structure (blue area in the figure) adopts a body element. The front end member is placed on a rotating roller with a diameter of 30 mm and a spacing of 100 mm. Above the middle position of the horizontal symmetry axis of the two rollers, a cylindrical indenter with a diameter of 30 mm is used to press the front member, and the downward displacement is 10 mm. The constitutive model of the front-end component was constructed with Mat_24 and the parameters of the constitutive model under complex stress were input, and the fracture curves of JC, MMC, and DIEM were, respectively, input in Mat_Add_Erosion. The indenter and roller are regarded as rigid bodies, and the Mat_20 rigid body material constitutive model is adopted.

#### 3.3.1. Calculation Result

**Figure 17:**
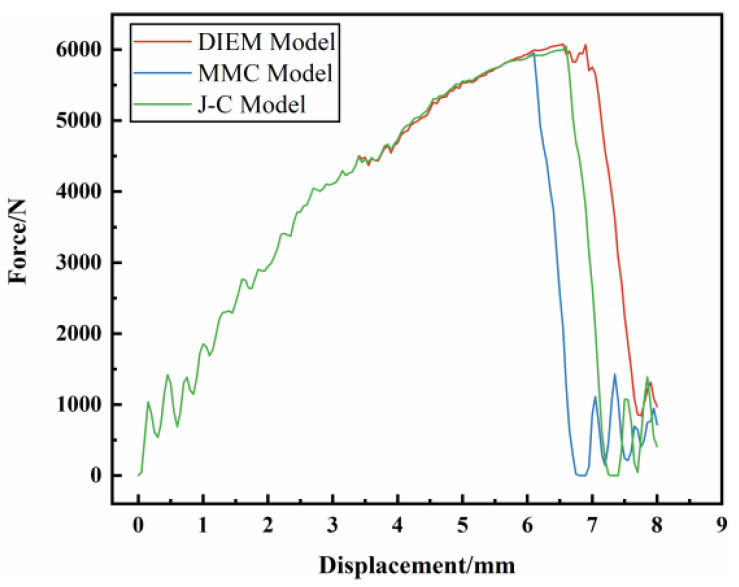
Three-point static simulation loading–displacement curve of front-end component.

Through simulation calculation, the three-point static loading–displacement curve of the front-end component is obtained, as shown in Figure 17, where the load is the contact force in the negative *y* direction between the front-end component and the indenter, and the displacement is the moving displacement in the negative *y* direction of the indenter. It can be seen from the figure that the JC, MMC, and DIEM fracture models are used to predict the fracture time of the AM60B die-cast magnesium alloy front-end members. The JC fracture model is the first to fracture, and the displacement of the indenter corresponding to the fracture time is 6.07 mm; the MMC fracture model is second, and the displacement of the indenter corresponding to the fracture time is 6.60 mm. The DIEM fracture model breaks at the latest, and the displacement of the indenter corresponding to the fracture time is 7.06 mm.

**Figure 18:**
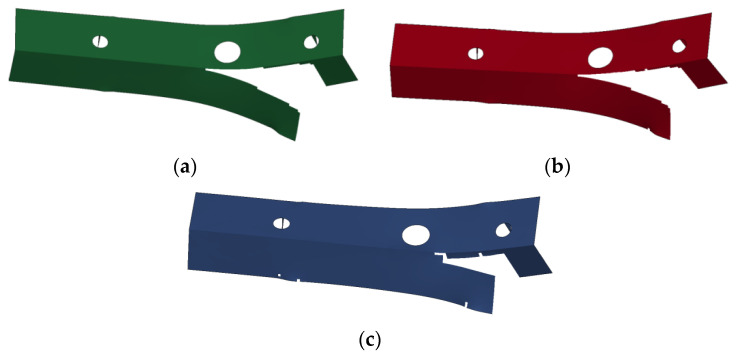
Three-point static simulation of fracture shape of front-end component: (**a**) JC model simulation result; (**b**) MMC model simulation result; (**c**) DIEM model simulation result.

The fracture shape predicted by three fracture models, JC, MMC, and DIEM, of the front end member of the AM60B die-cast magnesium alloy is shown in Figure 18. During the fracture process, the starting fracture point of the front end member is close to the contact position of the right roller. In the process of crack growth, the crack is first in the positive *y* direction and then in the negative *x* direction, and the shape after crack is similar, all of them are a reverse “L” shape.

#### 3.3.2. Experimental Verification

In order to verify the accuracy of the complex stress state constitutive model and fracture model parameters for predicting the quasi-static three-point bending deformation and fracture behavior of the AM60B die-cast magnesium alloy front end member, based on the simulation model conditions, the AM60B die-cast magnesium alloy front end member was used for actual three-point bending experiments. The experiment was performed on a universal tensile testing machine and repeated three times. The loading–displacement curve was output in the experiment, in which the load was the contact force between the cylindrical indenter and the front member, and the displacement was the downward pressure displacement of the cylindrical indenter. The load and displacement are collected by universal tensile testing machine.

**Figure 19:**
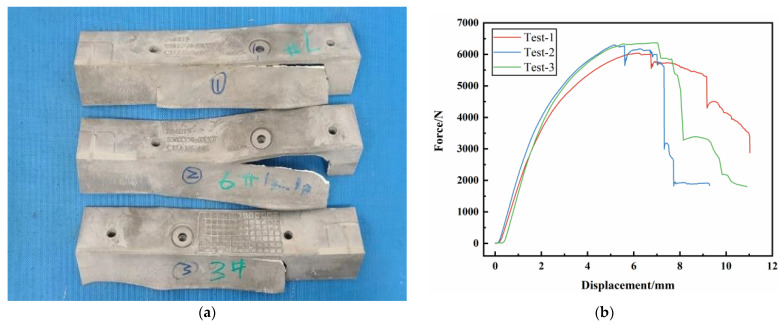
Three point static pressure test results of front-end components: (**a**) fracture shape of front end member; (**b**) loading–displacement curve.

Through experimental tests, the fracture shape and loading–displacement curve of the AM60B die-cast magnesium alloy front member obtained in three-point bending test are shown in Figure 19. During the experiment, the crack initiation point is close to the right roller contact position, and the crack shape is an inverted “L” shape. In the three experiments, the variation trend of loading–displacement curve is the same, but there are differences in the maximum load and displacement at breaking time. The maximum force in experiment 1, experiment 2, and experiment 3 is 5983 N, 6304 N, and 6358 N, respectively, and the displacement at breaking time is 9.32 mm, 7.32 mm, and 7.03 mm, respectively.

#### 3.3.3. Comparative Analysis of Simulation and Test Results

**Figure 20:**
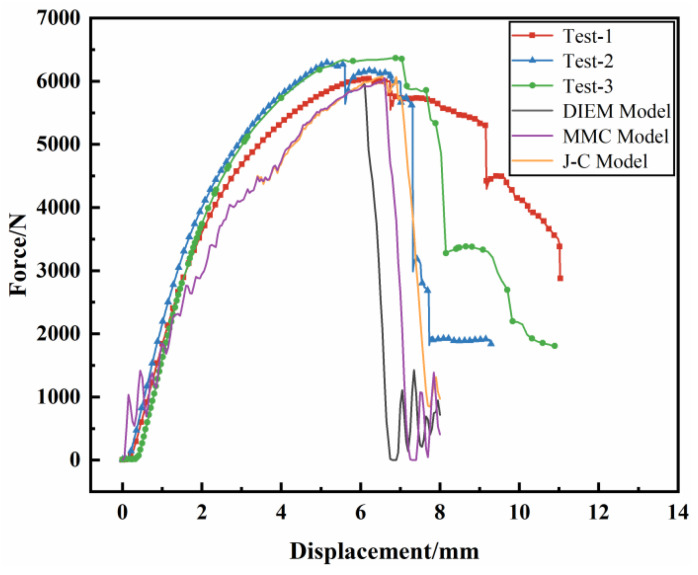
Comparison of three-point static pressure simulation of front-end component with experimental loading–displacement curve.

Compared with Figure 19 and Figure 20, it can be seen that in simulation and experiment, the crack initiation point of the magnesium alloy front component is the same, which is close to the right roller contact position. The crack shape after initiation is similar, all of which are an inverted “L” shape. The loading–displacement curve is compared as shown in Figure 18, and the change trend is the same. With the deformation of the front end member, the load increases first, and after the crack occurs in the front end member, the load decreases rapidly. In the simulation and experiment, the fracture time of the front-end member corresponds to the pressure displacement of the indenter, as shown in Table 7. It can be seen from the table that JC, MMC, and DIEM have different prediction accuracy for the fracture behavior of the front-end member, and the error of the fracture time is 21.56%, 14.71%, and 8.77%, respectively, among which the error of the fracture time of DIEM is the smallest. The prediction accuracy of fracture behavior is the highest. The results show that the parameters of complex stress state constitutive model and DIEM fracture model obtained in this paper have good accuracy and applicability.

## 4. Conclusions

Die-cast magnesium alloy has been well applied in automobile parts. The die-cast magnesium alloy parts will break in the process of automobile collision, and the stress state of the materials in the process of automobile collision is very complicated. In this paper, the plastic constitutive equation of AM60B under complex stress state is studied for automobile collision operation conditions. A new plastic constitutive equation suitable for automobile die casting magnesium alloy is established for the first time. Moreover, the accuracy of three models for predicting the fracture of die casting magnesium alloy is compared by simulation and experiment.

The main results of this study are summarized as following:

(1) The mechanical properties tests of the AM60B die-cast magnesium alloy sheet under four different stress states were studied. Based on the test results and isotropic constitutive model, the constitutive model parameters under uniaxial stress state and complex stress state were obtained using the parametric inverse method. The comparison of loading–displacement curves with inverse parameters shows that under the four stress states, the prediction accuracy of complex stress state constitutive model parameters on the deformation behaviors of the AM60B die-cast magnesium alloy sheet is better than 90%, indicating that complex stress state constitutive model parameters are more suitable for describing the deformation behaviors of AM60B die-cast magnesium alloy sheets.
(2) Based on JC, MMC, and DIEM fracture models considering stress states, the fracture behaviors of AM60B die-cast magnesium alloy sheets under four stress states, namely shear, tensile, R10 notch tensile, and cupping, were simulated and compared. The results showed that the comprehensive error of fracture time predicted by the DIEM fracture model is the smallest, and the prediction accuracy is greater than 95%, which indicates that the DIEM fracture model can accurately predict the fracture failure of the AM60B die-cast magnesium alloy sheet under various stress states.
(3) A three-point bending numerical model of the AM60B die-cast magnesium alloy front member was established, and the deformation and failure of the AM60B die-cast magnesium alloy sheet was predicted by using complex stress state constitutive model and the JC, MMC, and DIEM fracture models. Experimental verification was performed. It was found that the deformation mode and loading–displacement curve trend are the same in simulation and experiment. The crack initiation point, crack shape, and fracture time predicted by the DIEM fracture model are the same. The results show that complex stress state constitutive model parameters and DIEM fracture model can accurately predict the deformation and failure behavior of the AM60B die-cast magnesium alloy sheet. The results of this paper can provide some reference for improving the deformation and failure prediction accuracy of automobile magnesium alloy components under complex stress conditions.

## Figures and Tables

**Table 1:** Error of loading–displacement curve under different stress states.

Type of Test	Relative Error between Maximum Forces				The Maximum Relative Error of the Force under the Same Displacement			
	Curve 1	Curve 2	Curve 3	Absolute Mean	Curve 1	Curve 2	Curve 3	Absolute Mean
Shear test	5.99%	6.85%	6.72%	6.52%	3.68%	4.94%	3.09%	3.90%
Tensile test	0.22%	6.64%	3.14%	3.33%	4.36%	6.82%	3.23%	4.80%
R10 notch tensile test	7.74%	6.51%	6.72%	6.99%	7.87%	7.41%	6.72%	7.33%
Cupping test	17.97%	7.95%	12.59%	12.83%	18.60%	21.15%	13.02%	17.59%

**Table 2:** The errors of loading–displacement curves under different stress states are optimized.

Type of Test	Relative Error between Maximum Forces				The Maximum Relative Error of the Force under the Same Displacement			
	Curve 1	Curve 2	Curve 3	Absolute Mean	Curve 1	Curve 2	Curve 3	Absolute Mean
Shear test	1.92%	2.74%	2.62%	2.43%	5.07%	5.33%	3.51%	4.64%
Tensile test	8.43%	1.91%	5.25%	5.20%	8.59%	2.54%	5.43%	5.52%
R10 notch tensile test	1.62%	0.9%	0.17%	0.90%	1.73%	1.63%	0.34%	0.99%
Cupping test	12.27%	2.76%	7.15%	7.39%	12.90%	8.39%	8.37%	9.88%

**Table 3:** Fit of each strain rate equation.

Strain Rate Equation	1/s	10/s	200/s	500/s	1000/s	Average Fitting
Johnson–Cook	0.9983	0.9963	0.9891	0.9360	0.9610	0.9761
Modified Johnson–Cook	0.9817	0.9872	0.9712	0.9053	0.9905	0.9672
Cowper–Symonds	0.9985	0.9936	0.9909	0.9466	0.9849	0.9829

**Table 4:** The stress triaxiality and the critical fracture equivalent plastic strain of each specimen are calculated.

Type of Test	Stress Triaxiality *η*	Critical Fracture Equivalent Plastic Strain *ε_f_*
Shear test	0.01	0.134
Tensile test	0.333	0.118
R10 notch tensile test	0.386	0.128
Cupping test	0.666	0.454

**Table 5:** The fitting parameter values of each fracture model.

Fracture Model	Fitting Parameters				
JC fracture model	$D_{1}$	$D_{2}$	$D_{3}$		
	0.106	6.767	−17.842		
MMC fracture model	$C_{\theta }^{s}$	K	C	f	n
	1	669.973	142.197	0.076	0.447
DIEM fracture model	k	$e_{0}$	$e_{1}$	f	
	−3.970	0.317	0.007	0.593	

**Table 6:** The average error of fracture time predicted by each fracture model for each test.

Type of Test	Mean Error at Break Time		
	JC Fracture Model	MMC Fracture Model	DIEM Fracture Model
Shear test	8.64%	3.18%	4.11%
Tensile test	4.04%	4.45%	4.26%
R10 notch tensile test	9.08%	7.66%	6.23%
Cupping test	65.64%	62.26%	2.01%

**Table 7:** Prediction error of fracture behavior of front end members by fracture models.

Fracture Model	Prediction Error of Fracture Time of Front end Member			
	Curve 1	Curve 2	Curve 3	Average Value
JC	33.95%	17.08%	13.66%	21.56%
MMC	28.18%	9.84%	6.12%	14.71%
DIEM	23.18%	3.55%	0.43%	8.77%

## Data Availability

The raw/processed data required to reproduce these findings cannot be shared at this time as the data also form part of an ongoing study.
